# IL-37 isoform D downregulates pro-inflammatory cytokines expression in a Smad3-dependent manner

**DOI:** 10.1038/s41419-018-0664-0

**Published:** 2018-05-22

**Authors:** Mingsheng Zhao, Yulan Li, Chun Guo, Liyang Wang, Hongxia Chu, Faliang Zhu, Yan Li, Xiaoyan Wang, Qun Wang, Wei Zhao, Yongyu Shi, WanJun Chen, Lining Zhang

**Affiliations:** 1 Department of Immunology and Key Laboratory of Infection and Immunity of Shandong Province, Shandong University School of Basic Medical Sciences, 44 #Wenhua Xi Road, 250012 Jinan, China; 2 Department of pathogenic biology, Shandong University School of Basic Medicine Sciences, 44# Wenhua Xi Road, 250012 Jinan, China; 3 Mucosal Immunology Section, National Institute of Dental and Craniofacial Research (NIDCR), National Institutes of Health (NIH), 30 Convent Drive, Bethesda, Maryland 20892 USA

## Abstract

IL-37 is a new member of IL-1 family and possesses five different isoforms (named as IL-37 a–e). IL-37b has been demonstrated as a physiological suppressor of immune responses. However, the function of other isoforms remains unknown. Here, we show that IL-37d possesses anti-inflammatory roles both in vitro and in vivo. Firstly, IL-37d is expressed in peripheral blood mononuclear cells (PBMCs) and umbilical cords-derived mesenchymal stem cells (UCMSCs). Secondly, IL-37d overexpression markedly inhibits IL-1β-induced IL-6 production in A549 cells. Consistently, bone marrow-derived macrophages (BMDMs) from IL-37d transgenic mice express low levels of pro-inflammatory cytokines (such as IL-6 and TNF-α) following LPS stimulation, compared with those from wild-type mice. Furthermore, IL-37d transgenic mice produce less pro-inflammatory cytokines, and show much less degree of LPS-induced endotoxemia in vivo. Mechanistically, IL-37d interacts with Smad3 and promotes nuclear translocation of pSmad3. SIS3 (a specific Smad3 inhibitor) treatment completely blocks the inhibitory effects of IL-37d. Thus, our data indicate that IL-37d is a functional cytokine that negatively regulates pro-inflammatory cytokines expression in a Smad3-dependent manner.

## Introduction

Interleukin (IL)-1 cytokines family members play vital roles in innate immunity as the first defense line against pathogenic microorganisms and physical damage/stress. All IL-1 family members share a similar barrel structure and bind to Ig-like receptors^1–3^. IL-37, also called IL-1 family member 7 (IL-1F7), was discovered as a new IL-1 family member in 2000^4–6^. Human IL-37 gene consists of 6 exons. The exons 1–3 encode N-terminal sequences of IL-37 that possess a caspase-1 cleavage site and can be processed to its mature form. The exons 4–6 encode 12 putative β-strands, which are predicted to form the β-trefoil structure. IL-37 comprises five different isoforms, named as IL-37a–e, which are produced via alternative splicing^2, 3^. IL-37a (encoded by exons 3–6), IL-37b (encoded by exons 1, 2, 4–6), and IL-37d (encoded by exons 1, 4–6) contain the encoding sequences of 12 β-strands (exons 4–6), and are speculated as functional cytokines. However, IL-37c (encoded by exons 1, 2, 5, and 6) and IL-37e (encoded by exons 1, 5, and 6) are predicted to be nonfunctional because of the lack of exon 4 encoding for β-trefoil structure.

IL-37b is the longest transcript variant, which is encoded by five of six IL-37 exons (exons 1, 2, 4–6). Recently, it has been reported that IL-37b is detected in lymph nodes, placenta, colon, lung, kidney, testis, thymus, and uterus^7, 8^ and acts as an anti-inflammatory cytokine. IL-37b inhibits the expression of multiple pro-inflammatory cytokines, such as IL-1α, IL-1β, IL-6, and TNF-α^9–16^. Human IL-37b transgenic mice are resistant to LPS-induced endotoxin shock^12^, DSS-induced colitis^17^, surgical procedure-induced spinal cord injury^18^, and obesity-associated inflammation^19^. In addition, recombinant IL-37b protein attenuates endotoxemia^20^, rheumatoid arthritis^21^, invasive pulmonary aspergillosis^22^, systemic lupus erythematosus^23^, allergic airway inflammation^24^, ConA-induced hepatitis^25^, atherosclerosis^26^, myocardial ischemia/reperfusion injury^27^, and experimental psoriasis in mice^28^. IL-37b exerts its inhibitory roles in pro-inflammatory cytokines via IL-1R8 receptor-mediated extracellular pathway and Smad3-mediated intracellular pathway^12, 29^. Smad3 is a key intracellular signaling component of TGF-β signaling pathway, which can be phosphorylated after TGF-β receptors are stimulated by ligand. Smad3 is then translocated into nucleus to regulate its target genes. It has been reported that Smad3 can inhibit the activation of DCs and macrophages^30–32^. Although IL-37b has been identified as an important anti-inflammatory cytokine, the function of IL-37d remains largely unknown.

Here, we investigated the function of IL-37d using in vitro human IL-37d overexpression system and in vivo human IL-37d transgenic (IL-37dtg) mice. We found that IL-37d possessed anti-inflammatory roles similar to IL-37b. However, IL-37d exerted its inhibitory effects in a Smad3-dependent manner, but not by IL-18Rα-IL-1R8 (SIGIRR) receptor pathway. IL-37d interacted with Smad3 and promoted its nuclear translocation.

## Results

### IL-37d is expressed in PBMCs and UCMSCs

**Fig. 1:**
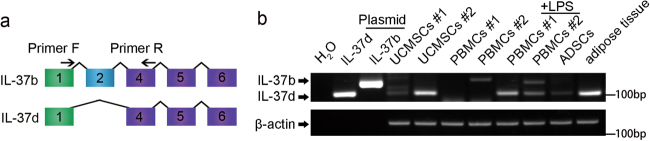
IL-37d was expressed in PBMCs and UCMSCs. **a** Diagrammatic sketch for design of IL-37 primers. Numbered boxes indicate individual exons of IL-37. **b** UCMSCs, PBMCs, ADSCs, and adipose tissue were from healthy donors. PBMCs were stimulated with or without LPS (100 ng/ml) for 24 h. The expressions of IL-37d and IL-37b were detected by RT-PCR. IL-37b and IL-37d recombinant plasmids were as positive controls

Previous research reported that IL-37d is expressed in bone marrows and testis^7^. To study whether IL-37d is expressed in peripheral blood, we isolated peripheral blood mononuclear cells (PBMCs) from six healthy volunteers to examine the mRNA expression of IL-37d by RT-PCR that differentiated IL-37d from IL-37b by the length of PCR products (103 bp for IL-37d and 166 bp for IL-37b), (Fig. 1a), because of lack of specific antibody to IL-37d. As shown in Fig. 1b, specific bands for IL-37d and IL-37b recombinant plasmids were detected and the PCR products were confirmed by DNA sequencing (Supplementary Fig. 1a and 1b). IL-37d expression was detected in LPS-stimulated or unstimulated PBMCs (Fig. 1b and Supplementary Fig. 1c). Furthermore, IL-37d was also detected in umbilical cord mesenchymal stem cells (UCMSCs) from healthy donors (Fig. 1b), adipose tissue-derived stromal cells (ADSCs) and in adipose tissue (Fig. 1b). These results indicate that IL-37d is constitutively expressed in PBMCs and UCMSCs.

### IL-37d inhibits the expression of pro-inflammatory cytokines in vitro

**Fig. 2:**
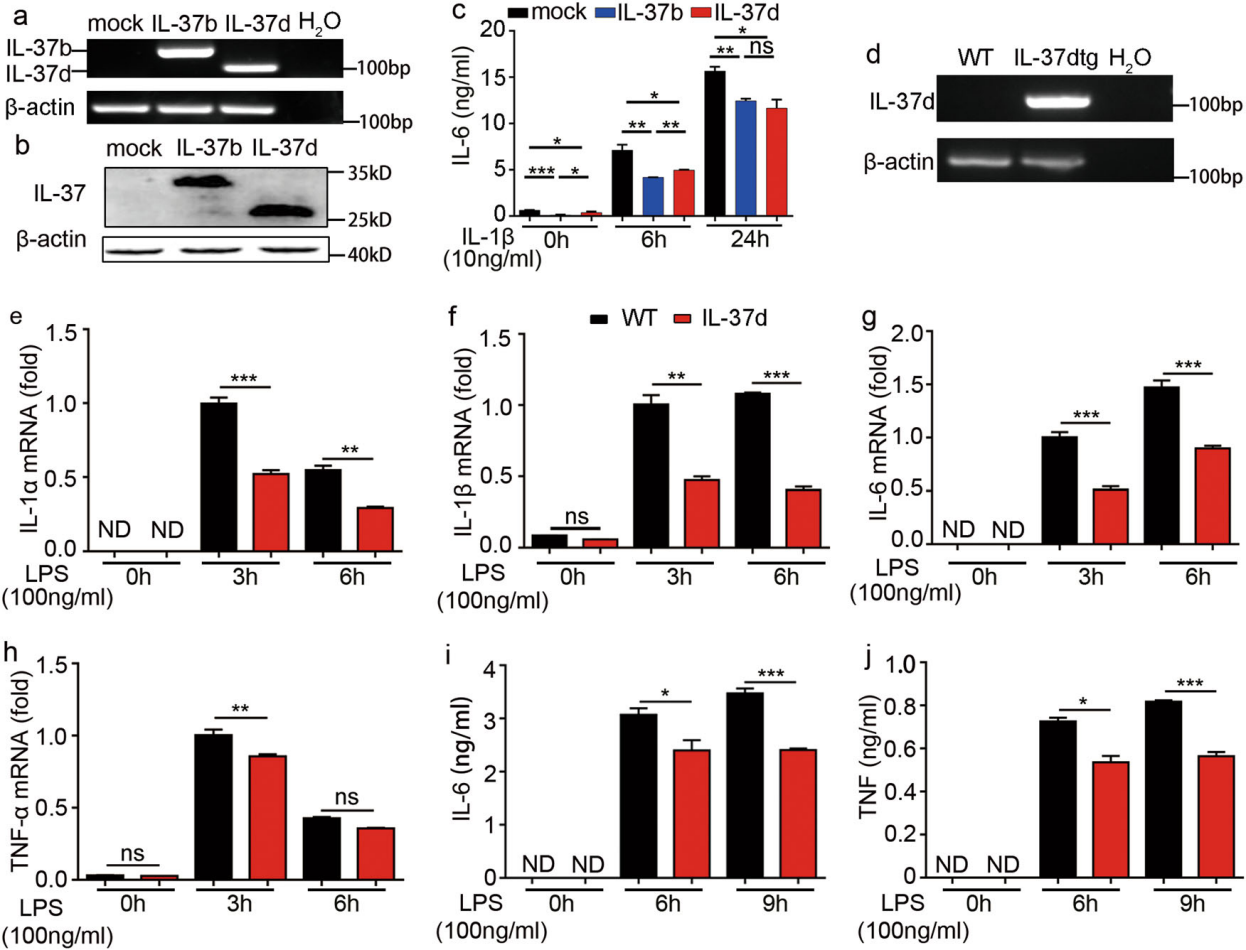
IL-37d inhibited pro-inflammatory cytokines expression. **a–c** A549 cells were transfected with the mock, IL-37b, or IL-37d recombinant plasmid, respectively, and stimulated with or without IL-1β (10 ng/ml) for the indicated times. The expressions of IL-37b and IL-37d were detected by RT-PCR (**a**) and western blot (**b**). The levels of IL-6 in the cell culture supernatants were determined by ELISA (**c**). The expression of IL-37d in bone marrow-derived macrophages (BMDMs) from wild-type and IL-37dtg mouse was detected by RT-PCR (**d**). BMDMs were stimulated with or without LPS (100 ng/ml) for 3 h, 6 h, or 9 h. The mRNA levels of IL-1α, IL-1β, IL-6, and TNF-α were determined by qRT-PCR (**e–h**) and the levels of IL-6 and TNF in the cell culture supernatants were determined by cytometric beads array (CBA) (**i**, **j**). ND, Not detectable. *P < 0.05; **P < 0.01; ***P < 0.001, ns, no significant difference. Data are shown as the mean ± SEM from three independent experiments

To investigate the functions of IL-37d in immune regulation, we transfected A549 cells with IL-37d recombinant plasmid (Fig. 2a, b) and examined the levels of pro-inflammatory cytokines produced by A549 cells following IL-1β stimulation. IL-1β induced large amounts of IL-6 expression (Fig. 2c), which was significantly suppressed by the overexpression of IL-37d or IL-37b in A549 cells (Fig. 2c). The data indicate that IL-37d negatively regulates IL-6 expression.

To further confirm the biological function of IL-37d, we generated IL-37d-transgenic (IL-37dtg) mice. IL-37dtg mice were back-crossed with wild-type C57BL/6 mice for six generations followed by crossing heterozygous mice for more than eight generations to generate homozygous IL-37dtg mice (Supplementary Fig. 2a and 2b). IL-37d mRNA expression was observed in bone marrow-derived macrophages (BMDMs) from homozygous IL-37dtg mice but not in BMDMs from wild-type mice (Fig. 2d). Importantly, decreased mRNA and protein levels of multiple pro-inflammatory cytokines, including IL-1α, IL-1β, IL-6, and TNF-α, were observed in LPS-stimulated BMDMs from IL-37dtg mice, compared with wild-type mice (Fig. 2e–j). Collectively, these data indicate that IL-37d inhibits the expression of pro-inflammatory cytokines.

### IL-37d suppresses pro-inflammatory cytokines in vivo and ameliorates LPS-induced endotoxemia

**Fig. 3:**
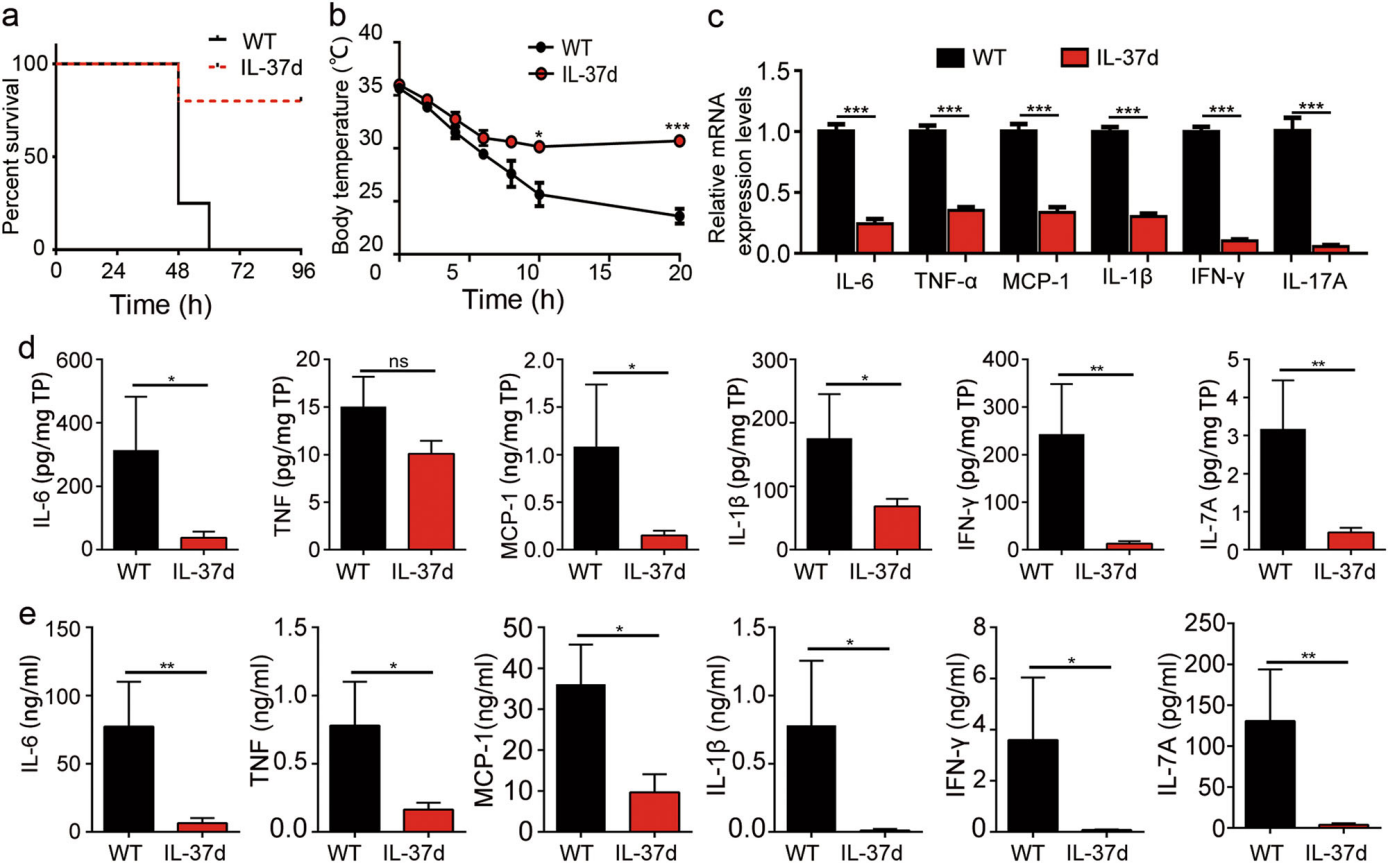
IL-37d ameliorated LPS-induced endotoxemia in vivo. **a** LPS (30 mg/kg) were intraperitoneally injected to IL-37dtg mice (*n* = 5) and WT mice (*n* = 4) and survival rate was analyzed. **b**–**e** LPS (10 mg/kg) were injected intraperitoneally to IL-37dtg mice (*n* = 13) and WT mice (*n* = 6) and then body temperature was measured at the indicated time points (**b**). The levels of IL-6, TNF-α, MCP-1, IL-1β, IFN-γ, and IL-17A in spleen were detected by qRT-PCR (**c**). The expressions of IL-6, TNF, MCP-1, IL-1β, IFN-γ, and IL-17A in lysates of spleen were measured by CBA. Results are presented relative to total protein (TP) (**d**). The levels of IL-6, TNF-α, MCP-1, IL-1β, IFN-γ, and IL-17A in serum were measured by CBA (**e**). *P < 0.05; **P < 0.01; ***P < 0.001, ns, no significant difference. Data are shown as the mean ± SEM

To investigate the regulatory roles of IL-37d in vivo, LPS-induced endotoxemia mouse model was used in wild-type and IL-37dtg mice. We found IL-37d expression was detected in spleen and bone marrow cells, as well as adipose tissue from IL-37dtg mice (Supplementary Fig. 2c–f). Both IL-37dtg mice and wild-type mice were intraperitoneally injected with 30 mg/kg LPS, while IL-37dtg mice showed significantly improved survival rate compared with wild-type mice (Fig. 3a). Further, after 20 h of intraperitoneal injection with 10 mg/kg LPS, hypothermia was severer in wild-type mice than that in IL-37dtg mice by measuring the body temperature (Fig. 3b). Furthermore, the expressions levels of IL-6, TNF-α, IL-1β, IFN-γ, IL-17A, and chemokine MCP-1 were lower in the spleens from IL-37dtg mice than those from wild-type mice, both at mRNA and protein levels (Fig. 3c, d). In addition, the serum levels of IL-6, TNF, IL-1β, IFN-γ, IL-17A, and MCP-1 were also greatly decreased in IL-37dtg mice (Fig. 3e). Collectively, these data indicate that IL-37d suppresses LPS-induced pro-inflammatory cytokines expression in vivo and ameliorates LPS-induced endotoxemia.

### IL-37d inhibits the production of pro-inflammatory cytokines independent of IL-1R8 receptor

**Fig. 4:**
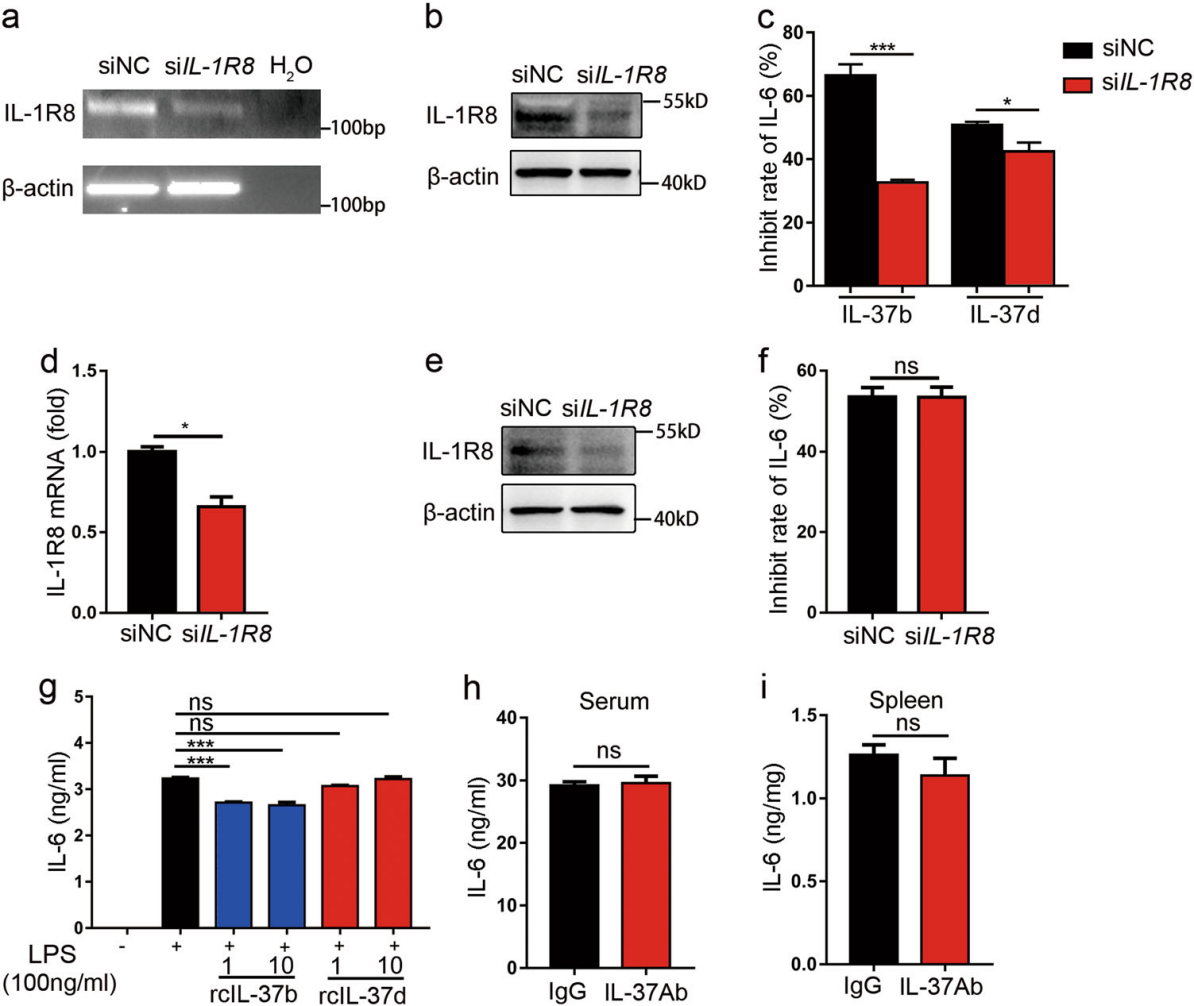
IL-37d inhibited pro-inflammatory cytokines expression in an IL-1R8-independent manner. **a**–**c** A549 cells were transfected with 100 nM of human siIL-1R8 or scrambled control for 24 h, followed by transfection of the mock or IL-37d plasmid, respectively, for additional 24 h and then stimulated with IL-1β (10 ng/ml) for 6 h; IL-1R8 knockdown efficiency was analyzed by RT-PCR (**a**) and western blot (**b**). The level of IL-6 in the cell culture supernatants was determined by ELISA and inhibition rate of IL-37d on IL-6 was calculated (**c**). **d**–**f** Peritoneal macrophages from IL-37dtg and wild-type mouse were transfected with murine siIL-1R8 or scrambled control siRNA for 48 h and followed by stimulation with LPS (100 ng/ml) for 6 h. IL-1R8 knockdown efficiency was analyzed by qRT-PCR (**d**) and western blot (**e**). Inhibition rate of IL-37d on IL-6 in the cell culture supernatants was determined by ELISA (**f**). **g** BMDMs from wild-type mouse were treated with increasing doses of recombinant IL-37b or IL-37d protein for 2 h and stimulated with LPS (100 ng/ml) for 6 h; The level of IL-6 in culture supernatants was determined by ELISA. **h**, **i** Monoclonal Ab against IL-37 (100 μg per mouse) or equal IgG2B as a control were injected intraperitoneally to IL-37dtg mice (*n* = 4 per group) for 3 h followed by LPS (5 mg/kg) intraperitoneal injection for additional 4 h. The levels of IL-6 in serum (**h**) and spleen (**i**) were measured by ELISA. *P < 0.05; ***P < 0.001, ns, no significant difference. Data are shown as the mean ± SEM from three independent experiments

It has been reported that IL-37b plays an anti-inflammatory effect in an IL-1R8 receptor-dependent manner^29, 33, 34^. To study whether the inhibitory roles of IL-37d are also IL-1R8 receptor dependent, we examined the effects of IL-1R8 knockdown on IL-37d function in both A549 cells and peritoneal macrophages. As shown in Fig. 4a–c, knockdown of endogenous IL-1R8 expression by IL-1R8 specific siRNA slightly decreased the inhibitory rate of IL-37d on IL-1β-induced IL-6 production in A549 cells, while it had no effect on mouse peritoneal macrophages (Fig. 4d–f), suggesting an independent role of IL-1R8. To further confirm this, we next compared the functions of recombinant IL-37d and IL-37b proteins in LPS-induced IL-6 expression in BMDMs. We found that IL-37d did not inhibit IL-6 in BMDMs, whereas IL-37b markedly suppressed IL-6 in BMDMs as expected (Fig. 4g). Furthermore, we found that the levels of IL-37d in mice were increased after LPS stimulation (Supplementary Fig. 3). However, IL-37 blockade via neutralizing antibody had no effects on IL-6 expression in both serum and spleen from IL-37dtg mice (Fig. 4h, i). Collectively, the data suggest that IL-37d suppresses the production of pro-inflammatory cytokines in an IL-1R8 receptor independent manner.

### IL-37d suppresses the production of pro-inflammatory cytokines in a Smad3-dependent manner

**Fig. 5:**
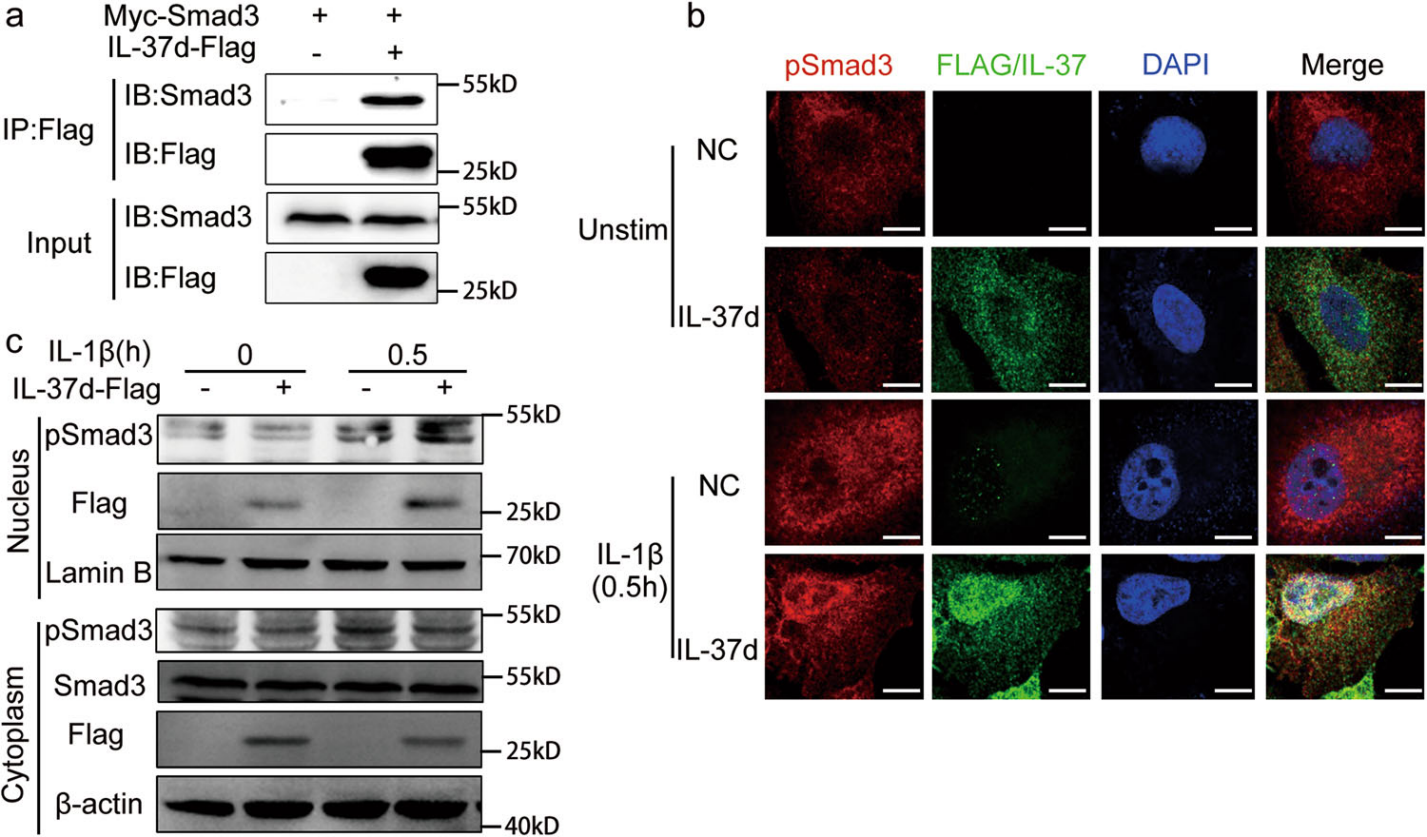
IL-37d interacted with Smad3 and promoted Smad3 nuclear translocation. **a** The recombinant plasmids containing IL-37d-Flag and Myc-Smad3 were co-transfected into HEK-293T cells and immunoprecipitation of IL-37d with Smad3 in cell extracts was performed using anti-Flag antibody and then followed by SDS-PAGE and immunoblotting with anti-Smad3 antibody. **b** A549 cells were infected with IL-37d-Flag recombinant or negative control lentivirus and then stimulated with or without IL-1β (10 ng/ml) for 0.5 h. Co-localization of IL-37d (Alexa-594, green) with phosphor-Smad3 (Alexa-647, red) and nuclei (DAPI, blue) was observed. Scale bar, 10 μm. **c** A549 cells expressing IL-37d-Flag were treated with or without IL-1β (10 ng/ml) for 0.5 h and then the expressions of pSmad3 and IL-37d in the nuclear and cytoplasmic extracts were detected by western blot. Data are representative of three independent experiments

IL-37b exerts its inhibitory roles in cytokine expression by both IL-1R8 and Smad3-mediated intracellular pathways^12, 29^. Thus, we next investigated whether the inhibitory effects of IL-37d were depended on Smad3. We first determined the interaction between IL-37d and Smad3 by co-immunoprecipitation. Flag-tagged IL-37d and Myc-tagged Smad3 plasmids were co-transfected into HEK-293T cells. As shown in Fig. 5a, IL-37d bound to Smad3. Next, the co-localization of IL-37d with phosphorylated Smad3 (p-Smad3) was detected by immunofluorescence. A549 cells were infected with Flag-tagged IL-37d lentivirus or control lentivirus. As shown in Fig. 5b, in the absence of IL-1β stimulation, the expression of pSmad3 and IL-37d were low in both nucleus and cytoplasm. After IL-1β stimulation, IL-37d was rapidly translocated to the nucleus and then promoted the nuclear translocation of pSmad3. Next, we examined the expression of pSmad3 and IL-37d in cytoplasmic and nuclear extracts of A549 cells by western blot. In consistent with the results from immunofluorescence, IL-1β stimulation promoted the nuclear translocation of both IL-37d and pSmad3 (Fig. 5c). Importantly, IL-37d enhanced the nuclear translocation of pSmad3 (Fig. 5c). Taken together, these results indicate that IL-37d interacts with pSmad3 and promotes its nuclear translocation.

**Fig. 6:**
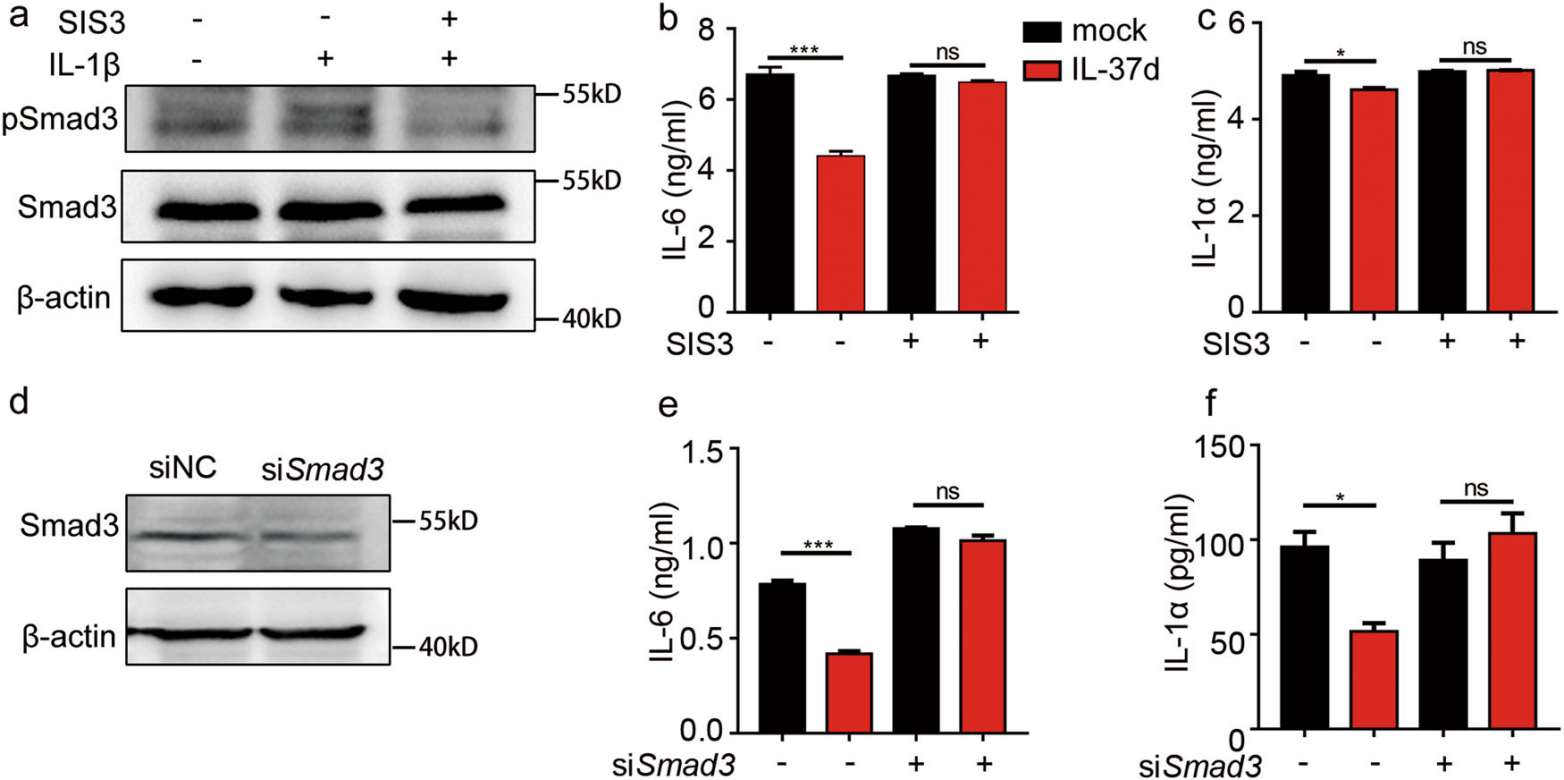
IL-37d attenuated pro-inflammatory cytokines expression in a pSmad3-dependent manner. **a** A549 cells pretreated with 2 μM SIS3 or the same amount of vehicle (DMSO) for 1 h were stimulated with IL-1β (10 ng/ml) for another 1 h. The expression of pSmad3 in whole-cell lysates was detected by western blot. (**b**, **c**) A549 cells transfected with IL-37d or mock plasmid were pretreated with 2 μM SIS3 or vehicle for 30 min and then stimulated with IL-1β (10 ng/ml) for 6 h. The levels of IL-6 (**b**) and IL-1α (**c**) in the culture supernatants were determined by ELISA. **d–f** A549 cells expressing IL-37d were transfected with 100 nM of human siSmad3 or scrambled control for 48 h and then stimulated with IL-1β (10 ng/ml) for 6 h. Smad3 knockdown efficiency was analyzed by western blot (**d**). The levels of IL-6 (**e**) and IL-1α (**f**) in the culture supernatants were determined by ELISA. *P < 0.05, ***P < 0.001; ns, no significant difference. Data are shown as the mean ± SEM from three independent experiments

To validate that the inhibitory effect of IL-37d is Smad3-dependent, a Smad3 specific inhibitor SIS3 was used to suppress Smad3 activity (Fig. 6a). As shown in Fig. 6b, c, the inhibitory effects of IL-37d on IL-1β-induced IL-6 and IL-1α expression were completely blocked by SIS3 in A549 cells. Furthermore, knockdown of endogenous Smad3 by Smad3 specific siRNA reversed the inhibitory rate of IL-37d on IL-1β-induced IL-6 and IL-1α production in A549 cells (Fig. 6d–f). Taken together, these data indicate that IL-37d suppresses pro-inflammatory cytokines expression in a Smad3-dependent manner.

## Discussion

IL-37 has five different splice variants (IL-37a–e). IL-37b has been suggested as a physiological suppressor of both innate and adaptive immunity. Recently, Yan *et al*. found the existence of common genetic variants of *IL-37b* in human^35^. However, the functions of other IL-37 isoforms remain unknown. Here, we show that IL-37d is a functional cytokine and plays anti-inflammatory roles. Mechanistically, IL-37d inhibits the production of pro-inflammatory cytokines via interacting with Smad3 and promoting its nuclear translocation.

IL-37d is discovered as an additional PCR product when amplifying IL-37 products from cDNA library of testis^7^. Furthermore, IL-37d is also detected in bone marrow using exon-specific primers, but not in lymph node, colon, lung, kidney, brain, heart, and placenta^36^. In our study, we found that IL-37d expression was detectable in some fresh isolated PBMCs (such as #NO 3, 6 cases), UCMSCs, ADSCs, and adipose tissue, suggesting IL-37d is constitutively expressed. However, it was undetectable or very low in some fresh isolated PBMCs and upregulated following LPS stimulation (such as #NO 1,2 cases). Previous studies have reported that mRNA of IL-37b was unstable and maintained in low levels in resting human PBMCs^6, 37^ or in monocytes^38^, even in murine RAW264.7 macrophages transfected by IL-37b recombinant plasmid with strong CMV promoter. It has been reported that an A-rich homology box within the open reading frame of IL-37b contributes to this instability^37^. Since IL-37d also contains the conserved homology box, we speculate that a similar mechanism of mRNA regulation as shown for IL-37b contributes to upregulation of IL-37d expression in stimulated cells. In addition, IL-37d is detected in mesenchymal stem cells, such as UCMSCs. Mesenchymal stem cells are involved in immune regulation. For example, graft-versus-host disease (GVHD) can be successfully treated by MSCs administrated^39, 40^. Thus, our results suggest that IL-37d is an important regulator of immunity. However, the significance and mechanisms of co-expression of IL-37d and IL-37b in cells remain to be further investigated.

Here we provide evidences to support that IL-37d is a functional cytokine. Firstly, IL-37d overexpression effectively inhibited IL-1β-induced IL-6 production (Fig. 2a–c). Secondly, BMDMs from IL-37dtg mice produced much less pro-inflammatory cytokines such as IL-6 and TNF-α, compared with BMDMs from wild-type mice (Fig. 2e–j). Furthermore, IL-37dtg mice produced lower levels of pro-inflammatory cytokines in an endotoxemia model (Fig. 3). Thus, our results suggest that IL-37d plays anti-inflammatory roles under stress situations.

IL-37b works as a cytokine via both intracellular and extracellular pathways. Initially, it is considered that IL-37b downregulates pro-inflammatory cytokines via the intracellular pathway. IL-37b translocates to nucleus following the cleavage process mediated by intracellular caspase-1^41^ and then binds to Smad3^12^. However, the co-localization between IL-37b and Smad3 is mainly observed in perinuclear and cytosolic regions^12^. SIS3 treatment or Smad3 knockdown partly reverses the inhibitory effect of IL-37b^12^. These data suggest that IL-37b can work in a Smad3-independent manner. Increasing evidence support that IL-37b also functions via extracellular pathway. IL-37b binds to the α-chain of IL-18 receptor (IL-18Rα)^6, 36, 42^. Recently, Nold-Petry et al. report that IL-37b binds to IL-18Rα and exploits IL-1R8 to form the tripartite complex IL-37b-IL-18Rα-IL-1R8, leading to the activation of multifaceted intracellular anti-inflammatory program^29^. IL-1R8 deficiency reverses the anti-inflammatory role of IL-37b in LPS-induced systemic endotoxemia model^29^. In addition, IL-1R8 is required for IL-37b to diminish allergic airway inflammation in mice^24^, inhibit inflammasome activation and disease severity in murine aspergillosis^22^, increase oxidative respiration, and improve exercise tolerance^43^. These data suggest that receptor-mediated extracellular function of IL-37b is functionally relevant. In our study, IL-1R8 knockdown has no significant effects on the inhibitory roles of IL-37d (Fig. 4a–f). IL-37d recombinant protein cannot suppress IL-6 expression (Fig. 4g) and IL-37 neutralizing antibody cannot reverse the function of IL-37d (Fig. 4h, i). However, SIS3 treatment and Smad3 knockdown completely reversed the inhibitory function of IL-37d (Fig. 6b–f). Thus, our data indicate that IL-37d suppresses the expression of pro-inflammatory cytokines in a Smad3-dependent manner. Smad3 belongs to receptor-regulated Smad (R-Smad) and is an intracellular signal transducer and transcriptional modulator activated by TGF-β and activin type-I receptor kinases^44^. Smad3 translocates to the nucleus to regulate gene expression^30^. Smad3 can interact with c-Jun and c-Fos to regulate the expression of a variety of different cytokines and chemokines^45–47^. Smad3, as an important intracellular transducer of TGF-β, can regulate the activation, proliferation, and differentiation of immune cells in both lymphoid and non-lymphoid tissues. Since TGF-β1 promotes the generation of FOXP3^+^Treg cells^48^, IL-37d may exert its anti-inflammation activity by upregulating Treg cells, which needs to be further addressed.

In conclusion, we find that IL-37d is a functional cytokine and plays important roles in controlling excessive inflammation, which is similar with IL-37b. However, IL-37d functions via a Smad3-dependent manner, which is different from IL-37b. Thus, our study identifies IL-37d as a regulator of immune responses and suggests it as a novel potential target for the treatment of inflammation-related diseases.

## Materials and Methods

### Recombinant plasmid and lentivirus

IL-37d cDNA was artificially synthesized and linked to a 3× Flag tag at its C terminus and cloned into pcDNA3.1-hisC plasmid. pcDNA3.1-hisC-IL-37b-3× Flag plasmid was generated by two times of reversed PCR using KOD-Plus-Mutagenesis Kit (TOYOBO, Osaka, Japan). First time, the PCR was done using primer (5′- AATTTTGTTCACACAAGTCCAAAGGTGAAGAACTTAAACCC and 5′ -GGCTTCCAGCCGGGTCTTCTAAGCAGCACTGGGGTTCATC) and pcDNA3.1-hisC-IL-37d-3×Flag plasmid as template to produce partial sequence of exon 2. For the second time, the PCR was done using primers (5′- CAAGCCTCCCCACCATGAATTTTGTTCACACAAGTCCAAGG and 5′-GGCCTGGTTCCAGGGGGCTTCCAGCCGGGTCTTCTAAG) and the product of first time PCR as template to generate recombinant IL-37b plasmid. For IL-37d recombinant lentivirus preparation, IL-37d was linked to a 3× Flag tag at its C terminus in GV358 lentivirus vector and prepared by Genechem, Co, Ltd (Shanghai, China). Briefly, recombinant IL-37d lentivirus (LV-IL-37d-EGFP) was produced in HEK-293T cells by cotransfection of the recombinant lentivirus vector with packaging plasmid using Lipofectamine 2000 (Invitrogen, Carlsbad, CA, USA). The titer of lentivirus is 5 × 10^8^ TU/ml. Human Smad3 cDNA was amplified from human lung cell line A549 by PCR and cloned in pCMV-N-Myc plasmid. All constructs were confirmed by DNA sequencing.

### Cell culture and transfection

A549 cells derived from human lung cancer and HEK-293T cells were obtained from China Center for Type Culture Collection (Wuhan, China). The A549 cells and HEK-293T cells were maintained in Ham’s F12/K medium and Dulbecco’s Modified Eagle Medium (DMEM) (Gibco, Invitrogen, Carlsbad, CA, USA), respectively, supplemented with 10% fetal bovine serum (FBS) (Gibco, Invitrogen, Carlsbad, CA, USA), 2 mM L-glutamine, 100 IU/ml penicillin, 100 μg/ml streptomycin at 37 °C in a humidified 5% CO_2_. For lentivirus infection, A549 cells (5 × 10^4^ cells/well) were seeded on 24-well plates for 12 h, infected by lentivirus (MOI = 20) with 5 μg/ml of polybrene for another 12 h according to the manufacturer’s protocol and then cultured in F12/K medium containing 10% FBS for 72 h. The efficiency of virus infection was observed under fluorescence-inverted microscope (Olympus, Japan). For transfection of plasmid, plasmid DNAs were transiently transfected into A549 cells or HEK-293T cells with Lipofectamine 2000 according to the manufacturer’s protocols (Invitrogen, Carlsbad, CA, USA).

### Preparation of PBMCs, UCMSCs, ADSCs, and adipose tissue

PBMCs were isolated from peripheral blood of six heathy volunteers by density gradient centrifugation using Ficoll-Hypaque (GE Healthcare Bio-Sciences Corp., Piscataway, NJ, USA). Adipose tissue was obtained from volunteer who accepted an abdominal operation (Qilu Hospital, Jinan, China). For ADSCs, the stem cell fraction was isolated from adipose tissue using type-I collagenase (Wako Pure Chemical Industries, Osaka, Japan). The study received ethical clearance from Qilu Hospital of Shandong University. Umbilical cords were obtained from healthy puerpera. The donors had no family genetic or cancer history. The donors’ sera were assessed to exclude HBV, HCV, HIV, EBV, CMV, and syphilis infection. Signed informed consent was obtained prior to delivery from all donors. UCMSCs were prepared as described in previous research^49^. Briefly, the umbilical cord was dissected with scissors into pieces approximately 1 mm^3^ in volume which then were enzymatically dissociated with collagenase type II at 37 °C for 1 h followed by digestion with trypsin at 37 °C for 30 minutes. After the tissue was filtered through a 200-mesh filter, the cells were plated in a cell culture dish with DMEM medium supplemented with 10% FBS. After incubation of the cells in a humidified atmosphere with 5% CO2 at 37 °C for three days, the tissue and non-adherent cells were removed by replacement of the fresh medium, and thereafter the medium was changed twice weekly. Once 70–80% confluence had been reached, the adherent cells were harvested for extraction mRNA. The study protocol was approved by the Ethical Committee of the Second Hospital of Shandong University. All subjects provided written informed consent and conformed to the principles outlined in the Declaration of Helsinki.

### RNA interference assay

Small interfering RNAs (siRNAs) were synthesized as following sequences: human IL-1R8: I, 5′ -AGUUUCGCGAGCCGAGAUCUU-3′, II, 5′-UACCAGAGCAGCACGUUGAUU-3′, III, 5′ -UGACCCAGGAGUACUCGUGUU-3′, IV, 5′-CUUCCCGUCGUUUAUCUCCUU-3′; human Smad3: 5′-GAUAAAGAAACCAGUGACCTT-3′; murine IL-1R8: 5′-GAUACAAACUCUUCCUAGATT-3′ and negative control: 5′-UUCUCCGAACGUGUCACGU-3′. The human siRNAs duplexes were transfected into A549 cells and murine siRNAs were done into mouse peritoneal macrophages using INTERFERin reagents according to the manufacturer’s instructions (Polyplus, Illkirch, France).

### Generation of IL-37d transgenic mice

Fertilized eggs from C57BL/6 mice were injected with the pIRES human IL-37d expression plasmid and implanted into C57BL/6 females by Cyagen Biosciences Inc (Suzhou, China) to generate founders, and male founders were mated with C57BL/6 wild-type females (Slaccas, Shanghai, China) for six generations. The genotypes of mice at 3–4 weeks of age were identified by PCR. PCR-negative littermates and wild-type mice were used as controls. All animal experiments were undertaken in accordance with the National Institute of Health Guide for Care and Use of Laboratory Animals with the approval of the Scientific Investigation Board of Medical School of Shandong University, Jinan, Shandong Province, China.

### Induction of bone marrow-derived macrophages

The bone marrow cells were harvested from femur and tibia of mice and then induced for macrophages in DMEM medium supplemented with 10% FBS in the presence of 100 ng/ml M-CSF (PEPROTECH, Rocky Hill, NJ) in humidified 5% CO_2_ at 37 °C for seven days.

### Endotoxic shock model and neutralizing antibody blocking experiment

For survival model, female mice aged 8–10 weeks were injected intraperitoneally with 30 mg/kg LPS (*E. coli* 055:B5, Sigma) and were monitored at indicated times. For endotoxic shock model, female mice aged 8–10 weeks were injected intraperitoneally with 10 mg/kg LPS (*E. coli* 055:B5, Sigma) and their body temperature were monitored by infrared electronic thermometer (DT-8806S, CEM, Shenzhen, China) at indicated times. Twenty hours after intraperitoneal injection of LPS, IL-37dtg, or WT mice were anesthetized and the serum and the spleen were obtained and used for analyzing cytokines. For neutralizing antibody blocking experiment, eight-week-old male IL-37dtg mice were intraperitoneally injected with 100 μg of IL-37 neutralizing antibody per mouse (MAB1975, R&D Systems) as described in previous research^50^. After 3 h, the mice received 5 mg/kg LPS via intraperitoneal injection, and after 4 h, the mice were anesthetized and the serum and the spleen were obtained and used for analyzing cytokines.

### IL-37d and IL-37b recombinant protein

Mature IL-37b recombinant protein was obtained from R&D systems (Cat.No.1975-IL). For mature IL-37d (21-197aa) recombinant protein preparation, IL-37d was cloned into pET-22b plasmid and expressed in Rosetta *E.coli*, and then purified by using Ni resin (TAKARA, Japan). The endotoxin was removed by ETErase^TM^ (Xiamen Bioendo Technology, Xiamen, China) and the level of endotoxin was measured by Chromogenic End-point Tachypleus Amebocyte Lysate (Xiamen Bioendo Technology, Xiamen, China). Purified IL-37d with low level of endotoxin (below 0.1 EU/mg) was resolved in phosphate buffer saline (PBS) and adjusted to the concentration of 5 mg/ml for use.

### ELISA

The concentrations of IL-6 were measured using ELISA MAX^TM^ Standard Sets, human IL-6 (Cat.No.430501) and mouse IL-6 (Cat.No.431301) (Biolegend, San Diego, USA) according to the manufacturer’s instructions. The concentration of IL-37 was measured by ELISA (Cat.No.88-52103-22, Thermo Fisher, Vienna, Austria). The concentration of human IL-1α was measured by ELISA (Cat.No. 70-EK101B2, MultiSciences, Hangzhou, China).

### Cytometric Bead Array (CBA)

Levels of IL-1β, IL-6, IL-17A, IFN-γ, TNF, and MCP-1 in the serum and spleen of mice were evaluated by Cytometric Bead Array (CBA) with Flex Set kit from BD Biosciences according to the manufacturer’s instructions using a FACSAria III flow cytometer (Becton, Dickinson and Company, USA). Protein in the spleens was extracted by tissue homogenate method and the concentrations of that were measured by BCA (Thermo, Rockford, USA).

### Immunoflurensence staining

Cells were cultured on Glass Bottom Cell Culture Dishes (NEST Biotechnology, Wuxi, China) for 12 h, fixed using Immunol Staining Fix Solution (Beyotime Institute of Biotechnology, China) for 10 min at room temperature, and blocked using Immunol Staining Blocking Buffer (Beyotime Institute of Biotechnology, China) for 1 h at room temperature. The cells were incubated in presence of pSmad3 primary antibody (1:100, Ser^423/425^, clone C25A9, Cell Signaling Technology, Danvers, USA) and Flag primary antibody (1:10000, Medical & Biological Laboratories, Co., Ltd. Nagoya, Japan) overnight at 4 ^°^C and then were washed three times with PBS followed by a 1 h incubation in the dark with the secondary antibodies coupled to Alexa Fluor 647 (Abcam, Cambridge, UK) or Alexa Fluor 594 (Abcam, Cambridge, UK), respectively. After another three washes with PBS, nuclei were stained by ProLong® Gold Antifade Mountant with DAPI (P36941, life technologies corporation, USA). The cells were observed under a confocal laser microscopy (LSM780, Carl Zeiss, Oberkochen, Germany).

### Immunohistochemistry

The paraffin slides were deparaffinized in xylene and hydrated in a graded ethanol series to distilled water. The slides were pretreated in citrate buffer (pH 6.0) in a microwave oven for 15 min. Next, the sections were rinsed in distilled water and treated with 3% H_2_O_2_ for 10 min. After rinsed in PBS, the slides were incubated for 1 h at room temperature with normal goat serum and subsequently incubated at 4 °C overnight with rabbit anti-IL-37 (Thermo Scientific, Rockford, USA) antibody at a dilution of 1:200. Then, Secondary staining was performed with HRP-conjugated anti-rabbit IgG using a MaxVsion Kit and 3, 5-diaminobenzidine (DAB) peroxidase Substrate Kit (Maixin Co., Fuzhou, China) followed by counterstaining with Mayer’s hematoxylin.

### RT-PCR and real time PCR

Total RNAs were extracted with TRNzol reagent according to the manufacturer’s instructions (Tiangen, Beijing, China), reversely transcribed into cDNA with PrimeScript™ RT reagent Kit with gDNA Eraser (TAKARA, Japan). The expressions of genes were detected by quantitative RT-PCR (qRT-PCR) using FastStart Universal SYBR Green Master (Roche Applied Science, Penzberg, Germany) on the Bio-Rad CFX 96 (Bio-Rad, California, USA) and the corresponding primer sequences are as follows: murine TNF-α: 5′-CCCTCACACTCAGATCATCTTCT-3′, 5′-GCTACGACGTGGGCTACAG-3′; murine IFN-γ :5′-ATGAACGCTACACACTGCATC-3′, 5′-CCATCCTTTTGCCAGTTCCTC-3′; murine IL-17A :5′-TTTAACTCCCTTGGCGCAAAA-3′, 5′-CTTTCCCTCCGCATTGACAC-3′; murine IL-6 :5′-CTGCAAGAGACTTCCATCCAG-3′, 5′AGTGGTATAGACAGGTCTGTTGG-3′;murine IL-1β:5′-GCAACTGTTCCTGAACTCAACT-3′, 5′-ATCTTTTGGGGTCCGTCAACT-3′;murine MCP-1:5′-TTAAAAACCTGGATCGGAACCAA-3′, 5′-GCATTAGCTTCAGATTTACGGGT-3′; murine IL-1α: 5′-CGAAGACTACAGTTCTGCCATT-3′, 5′-GACGTTTCAGAGGTTCTCAGAG-3′; IL-37dtg genotype identification: 5′-ACTTAAACCCGAAGAAATTCAGC-3′, 5′-GCCGACTCCAGCATGTTCC-3′; IL-37:5′- TGAACCCCAGTGCTGCTTAG-3′, 5′-CCCAGAGTCCAGGACCAGTA-3′; murine IL-1R8:5′-GTGACATGGCCCCTAATTTCC-3′, 5′- ATGCCAGACCATCTTTCAGCC-3′; human IL-1R8:5′-CACTGAAGTCTATGGGGCCTT-3′, 5′-ACGTTGAGACGGCACTTGAC-3′; murine β-actin 5′-TGCGTGACATCAAAGAGAAG-3′, 5′-TCCATACCCAAGAAGGAAGG-3′; human β-actin 5′-AGCCTCGCCTTTGCCGA-3′, 5′-CTGGTGCCTGGGGCG-3′.

### Western blot

Proteins from cells (30 μg) were separated on SDS-polyacrylamide gel and then transferred onto PVDF membranes (Millipore, Billerica, MA) after electrophoresis. Membranes were then blocked with 5% bovine serum albumin (BSA) in TBS containing 0.1% Tween-20 for 2 h and were probed overnight at 4 °C with primary antibodies followed by secondary antibody conjugated with HRP for 1 h at room temperature. Primary rabbit anti- IL-37 (IL-1F7) polyclonal antibody was obtained from Thermo Scientific, rabbit anti-Smad3 monoclonal antibody (C67H9) and phosphor-Smad3 (Ser^423/425^) monoclonal antibody (C25A9) were obtained from Cell Signaling Technology. Goat anti-Lamin B polyclonal antibody(C-20) was purchased from Santa Cruz. Rabbit anti-IL-1R8 polyclonal antibody was purchased from Proteintech. Mouse anti-β-actin monoclonal antibody and HRP-conjugated anti-goat antibody were obtained from ZSGB-BIO, Beijing, China. HRP-conjugated anti-murine and anti-rabbit IgG were purchased from Jackson ImmunoResearch (West Grove, PA, USA). Then membranes were visualized by ECL detection system (Sage Creation Science, Beijing, China).

### Co-Immunoprecipitation

Whole-cell extracts were lysed in IP buffer containing 1.0% (vol/vol) Nonidet P 40, 50 mM Tris-HCl, pH 7.4, 50 mM EDTA, 150 mM NaCl, and protease inhibitor ‘cocktail’ (Sigma). After centrifugation for 10 min at 14,000× *g*, supernatants were collected and incubated with anti-FLAG M2 magnetic beads (Sigma) for 6 h. Then beads were washed five times with IP buffer. Immunoprecipitates were eluted by boiling with 2 × LDS sample buffer (Thermo, Rockford, USA) and then analyzed by western blot.

### Inhibition of Smad3 activity by specific inhibitor

Smad3 specific inhibitor SIS3, (2E)-1-(6,7-Dimethoxy-3,4-dihydro-1H-isoquinolin-2-yl)-3-(1-methyl-2-phenyl-1H-pyrrolo[2,3-b] pyridin-3-yl)-propenone hydrochloride) (Biovision, Milpitas, USA) at concentrations of 2 μM was added in the culture system for 1 h to inhibit the activity of Smad3 with the initial cell density of 1.5 × 10^5^ cells/ml. Phosphorylated Smad3 was detected by western blot. The equal volume of DMSO was added to the cell culture system as control.

### Isolation of nuclear and cytoplasmic extracts

The nuclear and cytoplasmic extracts were isolated using an NE-PER Nuclear Cytoplasmic Extraction Reagent kit (Thermo, Rockford, USA) according to the manufacturer’s instruction. Briefly, the cultured cells were harvested and washed twice with cold PBS. The cell pellet was suspended in 200 μl of cytoplasmic extraction reagent I by vortexing. The suspension was incubated on ice for 10 min followed by the addition of 11 μl of a second cytoplasmic extraction reagent II, vortexed for 5 s, incubated on ice for 1 min, and centrifuged for 5 min at 16,000× *g*. The supernatant fraction (cytoplasmic extract) was transferred to a pre-chilled tube. The insoluble pellet fraction, which contains crude nuclei, was resuspended in 100 μl of nuclear extraction reagent by vortexing during 15 s, incubated on ice for 10 min, and then centrifuged for 10 min at 16,000× *g*. The resulting supernatant, constituting the nuclear extract, was used for the subsequent experiments.

### Statistical analyses

Data were analyzed by Student’s *t*-test or one-way ANOVA followed by LSD post hoc comparisons by the GraphPad Prism 6 (GraphPad Software, San Diego, CA, USA). All values in the text and figures represent the mean ± SEM, and *P* < 0.05 was considered significant.

## Electronic supplementary material

Supplemental information

Supplemental Figure 1

Supplemental Figure 2

Supplemental Figure 3
