# Supplementary material for: IL-37 isoform D downregulates pro-inflammatory cytokines expression in a Smad3-dependent manner
Source: Cell Death Dis. 2018 May 22;9(6):582. doi: 10.1038/s41419-018-0664-0 (PMC5964144; doi:10.1038/s41419-018-0664-0)
Supplement: Supplementary file 1 — Supplemental information [file 41419_2018_664_MOESM1_ESM.docx]

IL-37 isoform D downregulates proinflammatory cytokines expression in a Smad3-dependent manner

**Supplementary Figure 1. Sequencing data of IL-37 PCR products and expression of IL-37d in PBMCs.**

PCR was done by using IL-37d and IL-37b specific primers and PCR products were sequenced. **(a)** Sequence of IL-37d PCR product; (**b)** Sequence of IL-37b PCR product. **(c)** PBMCs were from healthy donors and were stimulated with or without LPS (100ng/ml) for 24h. The expressions of IL-37d and IL-37b were detected by RT-PCR. IL-37b and IL-37d recombinant plasmids were as positive controls.

**Supplementary Figure 2. Genotype** **identification and** **expression of IL-37d in IL-37dtg mice.**

**(a, b)** DNAs from mouse tail were obtained and then genotype of IL-37dtg mice was identified by PCR for 1st generation **(a)** and homozygous transgenic mice at 8th generation **(b)**. **(c, d)** Expression of IL-37d in spleen **(c)** and bone marrow cells **(d)** of IL-37dtg mice was detected by RT-PCR. **(e)** Expression of IL-37d in spleen from wild type and IL-37dtg mice which were given intraperitoneal injection of LPS (10mg/kg) or vehicle (N.S.). ND, Not detectable, *P<0.05. Data are shown as the mean ± SEM. **(f)** Expression of IL-37d in brown adipose tissue from IL-37dtg mice and WT mice which treated with normal chow diet (NCD) or a high-fat diet (HFD) for 24w was detected by immunohistochemical. Scale bar,20μm.

**Supplementary Figure 3. The concentration of IL-37d in serum of mice.**

IL-37dtg and WT mice were given intraperitoneal injection of LPS (10mg/kg) or vehicle (N.S.). The level of IL-37d in serum was measured by ELISA. ND, Not detectable. Data are shown as the mean ± SEM.
