# Supplementary figures and images for: IL-37 isoform D downregulates pro-inflammatory cytokines expression in a Smad3-dependent manner
Source: Cell Death Dis. 2018 May 22;9(6):582. doi: 10.1038/s41419-018-0664-0 (PMC5964144; doi:10.1038/s41419-018-0664-0)

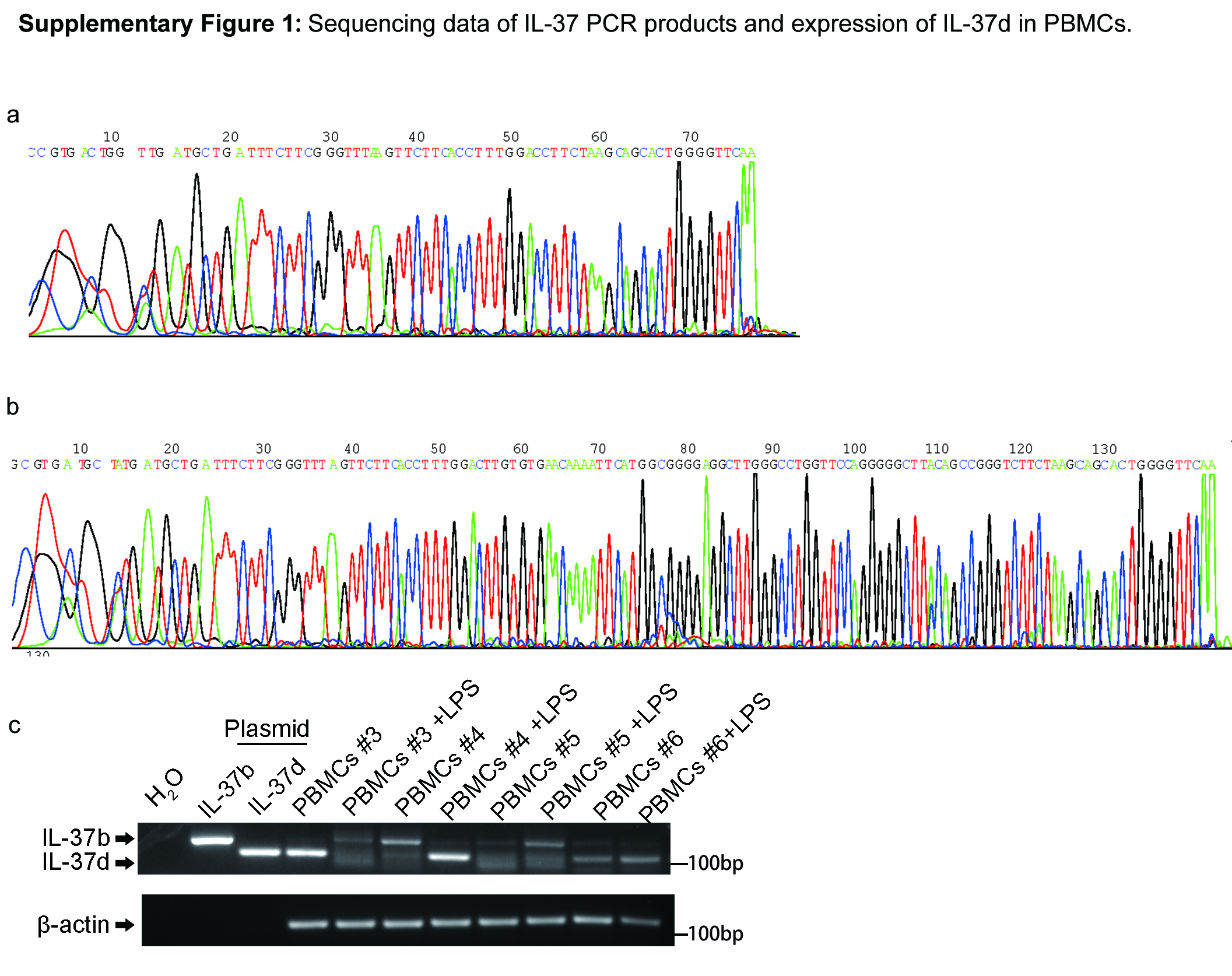

Supplement: Supplementary file 2 — Supplemental Figure 1 [file 41419_2018_664_MOESM2_ESM.tif]

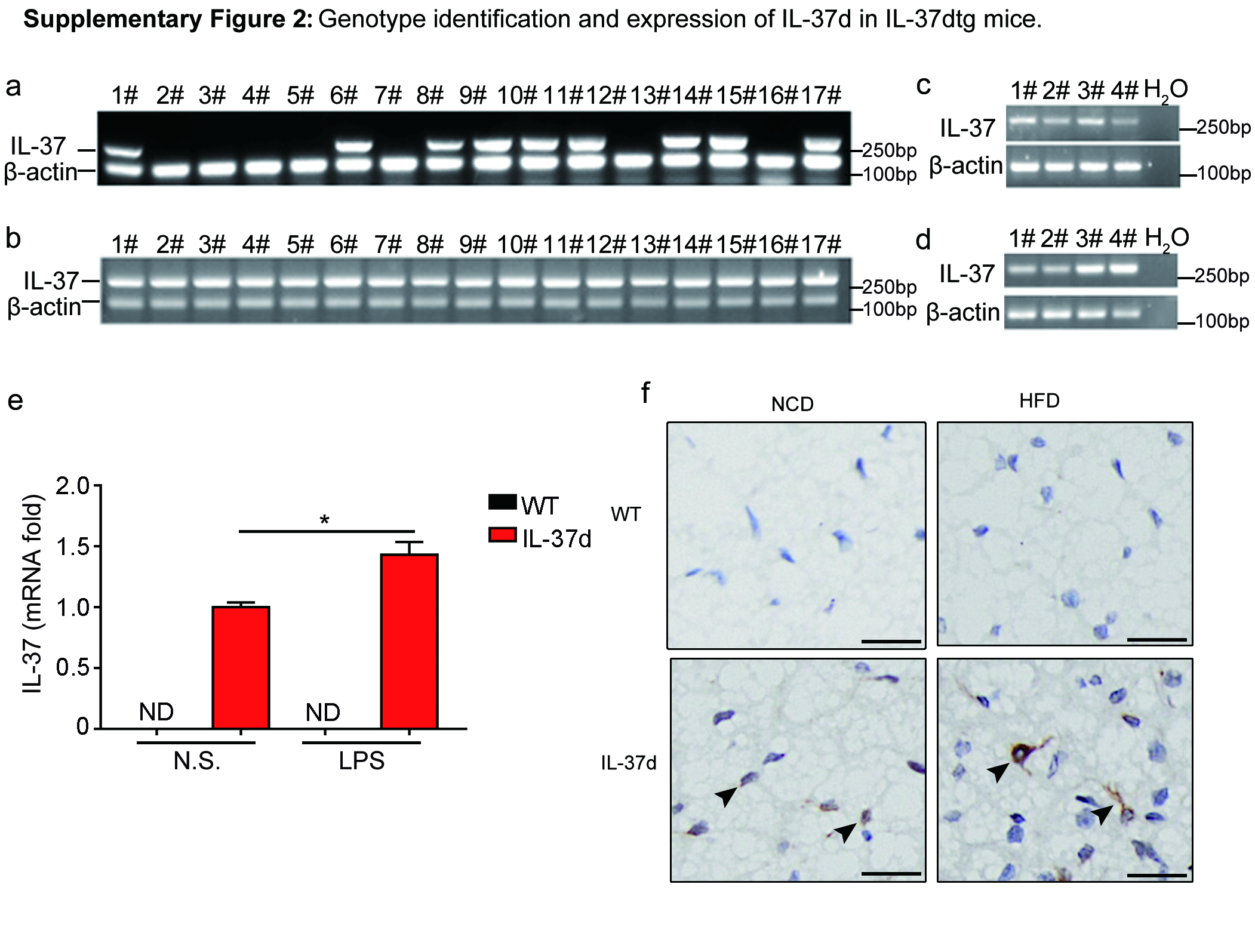

Supplement: Supplementary file 3 — Supplemental Figure 2 [file 41419_2018_664_MOESM3_ESM.tif]

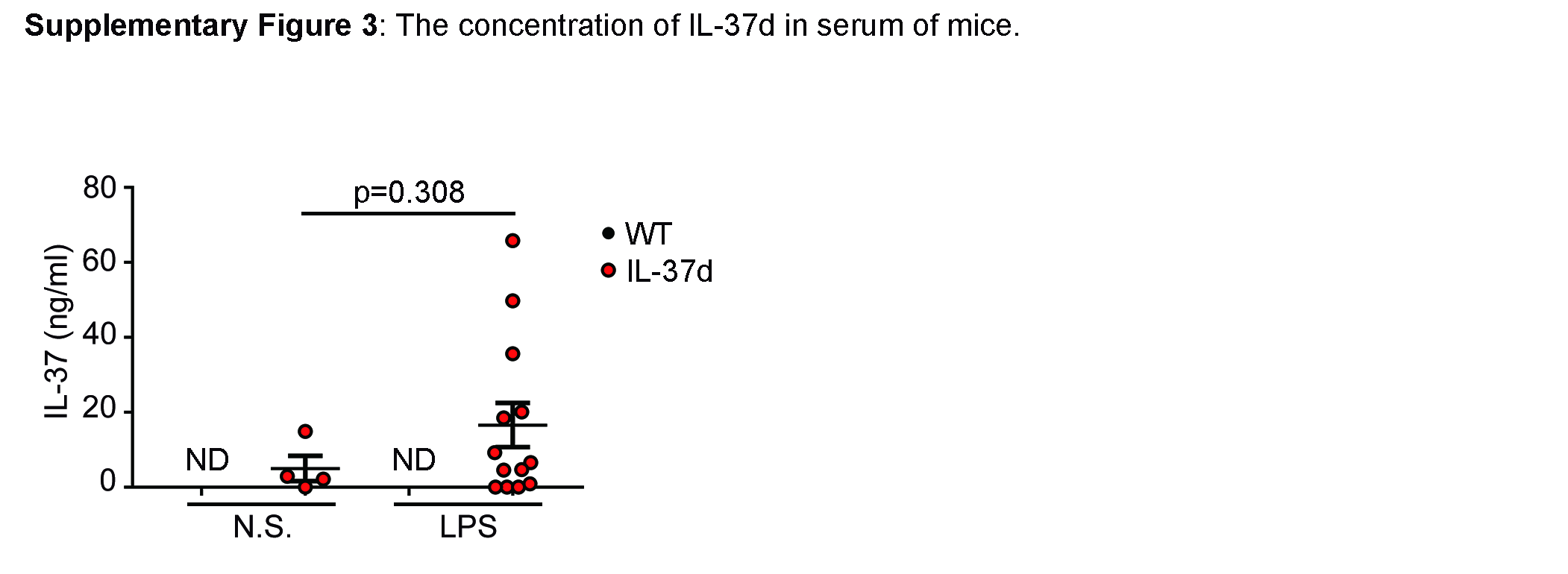

Supplement: Supplementary file 4 — Supplemental Figure 3 [file 41419_2018_664_MOESM4_ESM.tif]
